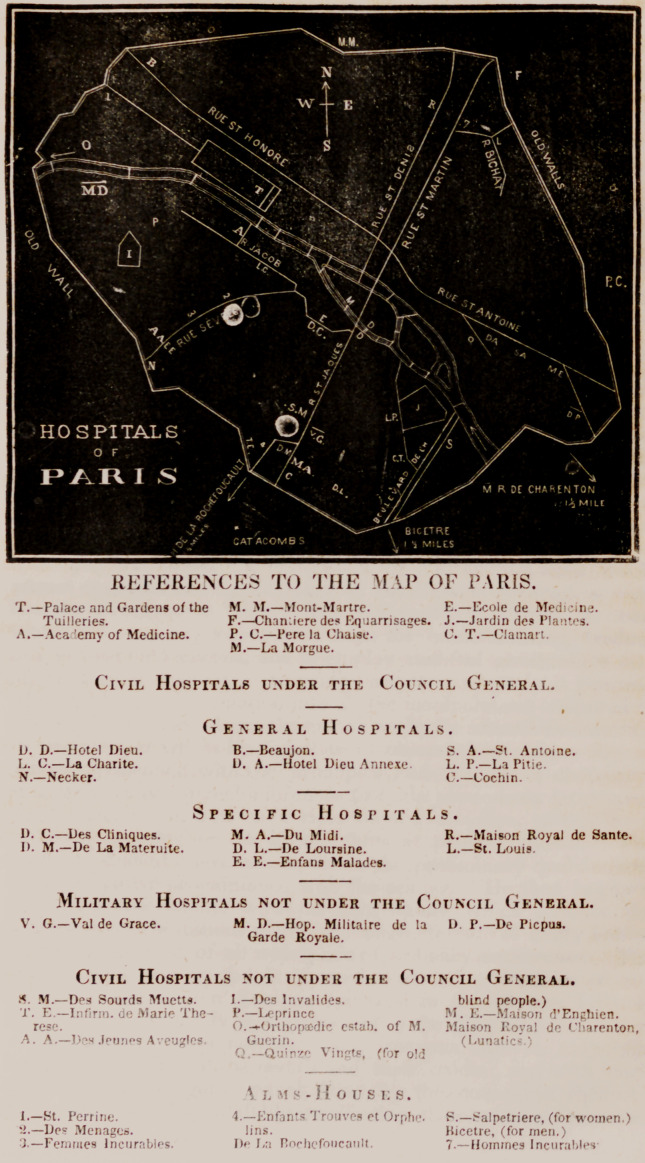# Notes of an European Tour

**Published:** 1846-12

**Authors:** F. H. Hamilton


ART. IV.—*Notes of an European Tour, by* F. H. HAMILTON, M. D.

PARIS.

To-day I visited Pere la Chaise, and walked several hours in its labarynth of aisles. The tombs which chiefly attracted my notice, were the tombs of the Cuvier family, of Dupuytren, of Beclard, Halle, Fourcroy, Hauy, the able Sicard and Laplace. Upon the highest point of ground, the spot which was once the site of a Jesuitical Convent, and the very focus of their power, I sat down to rest, and to enjoy at more leisure the broad and uninterrupted view of the cemetery not only, but of the whole valley of Paris. What a beautiful residence for the dead; and what a vast city of tombs has grown up here in 40 years. The rich dwell in "marble halls," in temples, mausoleums, sepulchral chapels, —beneath columns, alters, obelisks and huge pyramids, in the "upper town;" while the poor occupy narrow and crowded pits— "fosses communes" in the "lower town." They are removed every five years, when their lease expires, to make room for others, and to be sold for manure! for it is said these poor people being naturally gross, do great service in this way, and that but for the frequent revolutions in Paris, the land would be much less productive. Their *friends*, I see, hang no garlands on their cheap wooden crosses. At the gate I purchased a beautiful garland dedicated "a Ma Mere."

"La Pitie," is opposite the "Jardin des Plantes," where Beclard labored, and is at present famed as the work-house of Lisfranc, the coryphcus of uterus exsectors. But the "giant" is bending with the weight of years, and has at length given over his horrible operation of drawing out the uterus with iron claws, and cleaving it off like butchers meat. His patients got well, as his enemies soon proved, only to die—and he was compelled to be convinced.

His vast herculean frame, deeply carved features, slightly bending form, and frosted head, render him a distinguished mark, and contrast him strongly with all who gather about him. This morning I saw him make two double flap amputations : so far well enough, —but he did not forget the *charpie*, and this was sufficient to damn the whole operation. A hydrocele was laid open, and the large pouch crammed with charpie to the brim—"charpie! charpie! plus charpie!" And the pale sisters of charity are always at hand with "plus charpie," "charpie brute," "charpie rapee," "charpie en plumasseau," "charpie en bourdonnet," "charpie en tente," "charpie en meche," charpie en tampon," charpie en pelote!" Verily I believe no man in Paris would call himself decently, or honestly dead unless he had died of charpie—he might as well die without the last sacrament.

Berard is a slovenly, drawling Frenchman, and I followed his service just long enough to make this note:

"Salpetriere," (Saltpeter!) The great *female* Alms House and Lunatic Asylum of Paris.—It is a walled city, built of palaces, and contains a population of seven thousand inhabitants! But what a population! Lame, crooked, wasted, withered, old, drivelling, senseless, staring, fools and maniacs! It is a complete museum of all the crabbed, knotty and diseased specimens of the female sex that have ever existed. Yet Salpetriere has its church, its market, and its boulevard, where the *bon ton* saunter and gaze upon strangers.

M. Falret, a kind hearted and "fat man," accompanied me through the wards, and to the small detached houses occupied by the lunatics. These houses are one story brick buildings, each separated from the other by a considerable space, forming with the enclosure a large square, covered with a green sward. Here the worst of the maniacs live, each occupying one house alone; and they may shout and talk to their hearts content, without at all disturbing their neighbors. One woman was howling most dismally when M. Falret opened the door; she begged us to send her a Priest, for she said she had a Devil in her in the form of a puff of wind, who was holding her down in the flames of purgatory—she was burning constantly, and had been these four hundred years, but could not die—she wanted us to ask Louis Phillippe to have her guillotined. We promised to present her petition on the first opportunity.

M. Mitivie is one of the physicians. Pinel and Esquirol gathered from this field the chief elements and illustrations of their great doctrines of mental disease.

I have walked from morning until night through the "Jardin des Plantes," including all its various accessories of Menagerie, and Botanical, Meralogical, Zoological Galleries, &c. &c., and I cannot remember distinctly but three things; the bears climbing the poles, and the names of Buffon, and of Cuvier;—one thing more I remember, I was very tired when I had finished, and had sat down in an attitude partly reclining, upon a short bench beside one of the most retired walks, when a gentleman in blue, with white lace trimmings, a "gens d'armes" came toward me, and very civilly notified me to sit up, as it was "forbidden to recline in the kings garden." I begged pardon of the king and his bears, and went out.

"Clamart" was an old cemetery, upon the site of which are now erected Dissecting rooms. The dissecting rooms of Paris, are two, one adjoining the Dupuytren Museum, near l'Ecole de Medicine, and this, called Clamart. The rooms are all lighted from the roof. During the year about 4000 subjects are dissected here and at the other rooms; and as there are about 2000 Medical students on the average in Paris, if all were equally supplied, two subjects per year would be furnished to each student. They are furnished by order of the Government from the Public Hospitals. It is this enlightened policy which renders Paris the greatest school of anatomy in the world.

"Hopital du Midi." The theatre of M. Ricord's labours—the Americo-Gallic Hunter. I do not think M. Ricord is over 35 years old—he has a large head, and a plain face—with light complexion, and no one would take him for a Frenchman until he speaks, and then he is all fire and action—he dresses very plain, and is on the most intimate terms with patients, pupils and dressers. When in his wards, he is incessantly and rapidly engaged laughing, talking and *innoculating*:—now serious—now comic—now cauterizing his enemies — now his patients — now sending a stupid waiter away with a "sac-r-a"—now exhibiting a menagerie of paper animals, which Louis the old pet patient has been making the day before.

In the gallery of the Louvre, among many royal pictures hangs the portrait of M. Ricord. In the back-ground is seen dimly a speculum vaginæ "couchant,"—as a part of the proper blazonry of his escutcheon. Like the telescope of Galileo, it is the great instrument of most of his discoveries.

The Hopital du Midi contains 450 beds, and receives on the average about 3000 patients. It was originally a convent for capuchin friars. "To what base uses do we come at last!"

"La Loursine" is not far off, and is devoted to females affected with syphilis; it has on the average 2000 patients per year, of whom about 1 in 40 die; while at the Hopital Midi not more than 1 in 200 die! So much progress has the disease usually made among the females before they are admitted.

"Come," said my friend H——, the other morning as we left the clinique of M. Ricord, "let us go to the old church of St. Medard"—"what church is that?" said I, "for I have seen old churches enough, and would not now go ten feet to see the oldest, and most worm-eaten church in christendom." "Church of St. Medard!" said H——, with an expression of surprise at my ignorance or apathy, "why, it is the *fons et origo* of animal magnetism!" The church in which the famous priest called "Abbe Paris, the Jansenist" is buried, around whose tomb such curious pranks were exhibited by the convulsionists." "Oh! the convulsionists of St. Medard," said I, "then we will go."

The church itself looks as if it had come together in one of these Jansenic exstacies—Gothic, Doric, Corinthian, with a tall Dutch spire! "Come old man tell us all about it; where is the Abbe Paris buried?" "Cette eglise est tres ancienne Monsieur"—Yes, but where is the tomb of Paris? "Oui Monsieur c'est le plus ancienne a Paris---voici le tombeau de Nicole ici, et celui de Patru la." "Who cares for Nicole or Patru, we inquire only for the tomb of Paris the priest." "Ah Monsieur, St. Paris, St. Paris—n'est pas ici—c'est dans le cimetiere—c'est perdu tout a fait, tout a fait." But the church stands, in which these fanatics assembled during more than 12 years, and where were enacted scenes which rival in marvellousness, obscenity and absurdity, any thing which mesmerism has since disclosed. The subjects were uniformly *females,* and they came to the sepulchre of the sainted Paris for a cure for their various maladies, and a miracle was the result. "Les Soeurs," as they were self styled, who were under treatment, declared themselves totally deprived of all ordinary sensibility, and falling upon their backs, they would beg to have large paving stones cast upon them, and call upon stout men called "Freres," to stand and jump upon them; and they would cry out when the blows were suspended, "encore, mon cher Frere, encore." At last in 1732, the public authorities interfered, and the persecuted convulsionists suspended the following notice over the church door:

"De par le Roi, defense a Dieu,

De faire Miracle en ce lieu."

"Hopital du Val de Grace," attached to which is the church, erected by Ann, of Austria, in fulfillment of her vow for an heir, and in which still lies inurned the heart of the royal foundress. The hospital itself was once the fashionable convent for noble French ladies. Half the hospitals in Paris were, before the revolution convents and monasteries.

Vis-a-vis the Hopital du Midi, is the "Lying-in-Hospital," under the care of Moreau, P. Dubois, Madame Charriere, &c. No male pupils are admitted; but it educates annually about 60 girls, as sages-femmes. When a woman has been confined, she may take her child, or not as she chooses—if she refuses, it is sent to the Foundling Hospital, which is close by.

Look at this! In 1841 the births in this city were 30,000, of whom about 5000 were born in the hospitals, and about 10,000 were illegitimate—one half born honestly! 15,000 infants are constantly out at nurse in the country from the Foundling, and the Lying-in-Hospitals! Cost of foundlings per annum to the Government, $500,000! In 1841 the deaths were 26,000, of whom 10,000 died in hospitals, and 3,07 were exposed at the Morgue. There are about 70,000 paupers, and 20,000 foundlings constantly supported by Government, and on the average about 70,000 patients enter the various hospitals each year, all of which are sustained by the State! The hospitals cost $3,000,000 per year! Mean duration of life in Paris is 33 years; and families constantly residing in Paris become extinct after a few generations.

The "Infirmary of Marie Therese," is for sick ecclesiastics and ladies. The "Hopital Cochin" was founded by a Priest of that name. Both of the above are small.

Here we are after a long walk at the lodge of the beautiful hospital founded by Louis XVI. at the suggestion of Madame Necker, wife of the celebrated Minister; called from this circumstance "Necker." We have come all this way three hours before breakfast to see Civiale operate for stone. "La Suisse" says we are half an hour before the time, but we can go in. The calculous patients, six or seven in number, are in a ward by themselves, and they chat and laugh with us in a manner which shows clearly enough how little they fear the morning drill. Here comes M. Civiale at 7 o'clock precisely; he is about medium size, and a little corpulent, with a large plain, but intelligent and honest face; he is talking familiarly with my friend Dr. W. of Boston, and showing him a new lithontriptor which he has just finished. Every one bows and doffs his cap as he passes, and M. Civiale smiles and bows most unaffectedly in return. "Messieurs"—we all crowd about him, "this is a very bad stricture—the man thought he had stone, but he was mistaken, and we shall introduce this *sonde conique*," and in it goes with a slow, steady hand—the fellow complains, but it is too late, for the *sonde* is up to the hilt, and so steadily did it enter, that I would not have believed the fellow had a stricture, had I not seen an interne take hold of it to pull it out, and it was wedged in as with a hammer.

But his operations for stone are unparalleled; perfectly artistic; the highest excellence of surgical skill. We took our seats in a small amphitheatre, and a table was prepared in the arena with folded blankets, &c. The first patient was a man about 40 years old; he took his first setting just a week since, and M. Civiale then removed a large amount of soft stone. During the week he has passed one piece, which was sent around the room, about the size of a filbert, and very irregular. M. Civiale examined him, and found the stone was all gone. He told him to go home; live on light diet for a few days, and ride in an omnibus every day. The fellow went out looking as if reprieved from a sentence of death.

The next case is a lad, aged about 18. This is his second sitting also; the first a week since—the usual interval which Civiale gives, but longer if necessary. (Half an hour previous to each sitting a large wax bougie is introduced and suffered to remain until the operation is commenced.) The lad mounts the table laughing— Civiale withdraws the bougie and introduces a large catheter, and injects the bladder moderately with warm water; then withdraws the catheter and carries in carefully the lithontriptor of Heurteloup, modified. Like an honest man, he has hung his own original fiddle-string instrument upon the wall, and uses alone that of his rival. Yet no man in the world possesses the skill of Civiale in this operation. The stone in this case is hard and pretty large. The operator seizes it rapidly and breaks it three times in succession and then withdraws the instrument. The time occupied is about 40 seconds, during which the patient has not evinced the slightest pain. Patient now stands upright against the wall, and Civiale passes a catheter again, and immediately throws in with considerable violence a larger quantity of warm water, which the patient expels by the catheter. This part of the process provokes some complaints, and the only complaint he has yet made. A considerable quantity of reddish sand passes with the urine and is exhibited.

The third case is an old man, who is half frightened out of his wits, for this is the first operation. Civiale breaks the stone eight or nine times, and each time the old man hears it crack and cries out, but confesses as often that it did not hurt. When all is done, Civiale makes an examination and pronounces him completely quarried—not a fragment remains.

The fourth case is too irritable—must wait another week—and this completes the morning service. I am charmed with Civiale and shall visit him again.

M. Guerin lectures every Wednesday, from 10 to 12 A. M., on Deformities, at the "Hopital des Enfans Malades," a building connected with the Hopital Necker. His private institution, called "La Muette," is at Passy, just out of the old walls and near the

Bois de Boulogne. His advertisements produce a suspicion that he is an empiric, and his appearance confirms the suspicion. He has a stupid, but conceited face, and his remarks to the patients and to the few students who attend his lectures, at the hospital of sick children, are eminently quackish. I have visited his hospital and his cliniques frequently, and although he has many cases which are exceedingly interesting, and his diagnosis and treatment are sufficiently correct, yet there is in everything which he says an air of positive assertion and a self-glorification which smacks too plainly of charlatanism.

"Hotel des Invalides," founded by Louis XIV in 1670, covers sixteen acres of ground and encloses fifteen separate courts—a vast and superb edifice. Soldiers who have served 30 years in the regular army are entitled to its privilegs; also all who are disabled by wounds. They are boarded, lodged and clothed, and "vin ordinaire" is furnished at every meal. If a man has lost one leg he is allowed the cost of a shoe in money, and if two legs he receives double the amount. They have a large library, and live like gentlemen altogether. Napoleon is buried in the chapel of this hospital.

"Mon Dieu! Mon Dieu!" screamed a pretty little grisette, whom I had just passed on the "trottoir" of Rue St. Martin, tete-a-tete with a flash looking young Frenchman. "Qu' y a t' il maintenant" said I, turning quickly on my heel. "Mon Dieu! Mon Dieu! il est tue." The poor fellow fell like lead, and the grisette and myself lifted him together. Some careless femme de chambre had thrown a fragment of hard plaster from a fourth story window, which struck the Frenchman on the sinciput, making a hole through his hat and scalp, and rendering him completely senseless. He was sadly begrimmed with mud, plaster and blood. By a sort of natural surgical impulse I thrust my finger into the wound and immediately assured the grisette, who had never ceased screaming "il est tue—il est tue—Mon Dieu—Mon Dieu," that the bone was not broken and he would soon recover. "Oh Monsieur, vous etes tres obligeant, mais il est couvert de platre et de boue." "Ah true," said I, "but that is not half as bad as I was served yesterday when passing through la Cite, where I had a whole plate of fish scrapings thrown upon me." "Oh goujat!" said she, smiling kindly. Just here a policeman interfered, and directing the crowd "laisser" he lifted the Frenchman into a "voiture de remise" and with the afflicted grisette, who refused to be left, they drove off towards Hotel Dieu.

I was on my way to the Hospital of St. Louis, and as it was a walk of a mile or more, and this little accident had detained me some, I called a cabriolet and saying to the "cocher" to drive by way of Porte St. Martin to Hopital St. Louis, I took my seat. "*Sixty* sous" said the "cocher," as I handed him thirty at the gate of St. Louis. "How is that," said I, with a look of choler, to convince him that I had been in Paris too long to be practised upon, "I will report you to the "Poste." "Two drives Monsieur," said "cocher" very cooly, touching his hat and bowing "a la mode," "you stopped at Porte St. Martin." "You rascal, only one minute—scarcely that, to look at the arch." "True Monsieur, but it is two drives, thirty sous a drive." The argument was plain, and so, biting my under lip by way of a sedative, I gave him the whole. "In a citadine" said I to myself, "it would have cost *six* sous." "Troi sous pour boire," said the imperturbable "cocher," again touching his hat and bowing a foot lower than before. "A plague on your 'pour boire'" I exclaimed, in a violent pet, and thrusting my purse into my pocket with such force as to tear it an inch or more, I left him abruptly. "Who cares for the rent," said I, "I can mend it myself."

St. Louis dates its foundation from the pious king, St. Louis—some say it is much older, perhaps it is. It receives annually from 8000 to 9000 patients, of which the principal part have diseases of the skin. Cazenave, and Lugol of tincture notoriety, are in this department; but it has also a surgical service in which M. Jobert is most distinguished. Jobert is handsome, agreeable and not old, and has a great reputation for skill in the treatment of female maladies. The hot iron and nitric acid are his almost universal remedies for ulcers, tumors, &c., of the os tincæ. I have seen him apply the hot iron, through a large speculum, at least twenty times, and the patients are certainly generally relieved, if not cured. Jobert amputates with flaps, and, in spite of French prejudice, attempts union by adhesion. Yet he has not been very successful, if his enemies are to be believed, many of his patients having died of hospital gangrene. Jobert admits that some have been lost by this occurrence, but declares that the proportion so lost is smaller than when the old granulating system was practised. The truth is that his hospital has the most unfortunate location of any in Paris, being close by that incomparable depot of filth and ordure, Montfaucon; where, in immense basins, covering several acres, is gathered together the contents of all the sewers and closets of the city; and from which arises constantly a most intolerable stench, infecting the air of all this neighborhood, and, indeed, when the wind is favorable, of the whole of Paris. And cases of hospital gangrene are often to be seen in St. Louis, arising, as we believe, from this cause, among every variety of wounds. There is now a poor woman here who was slightly burned a few days since—hospital gangrene has occurred and she will probably die.

M. Jobert's practice in fractuie is original. If it is a broken thigh, he extends the patient on a mattrass, and placing a band in the perineum, secures him first to the head board, then three short straps are passed from a sandal on the foot to the foot board; these are buckled very moderately, and constitute all of the extending means employed. Three or four broad bands of roller are now laid across the leg at different points and tied under the bed, and these constitute all the lateral retentive means! The whole of the treatment is no more and no less than the above. When the fellow moves his leg M. Jobert scolds him—and when it is deformed or shortened, he says "the limb would have done well but the knave would not keep quiet." In a similar way he treats almost every fracture. For varicocele I have seen him operate by introducing five hare-lip pins under the veins at intervals of an inch, and winding the hare-lip suture across the opposite ends of each. It is followed immediately by a slight congestion of testis, but Jobert has never seen any worse consequence than a slight abscess. For an artificial anus he operated sucessfully "par torsion du lambeau"—closing the new wound with hare-lip sutures and securing the flap with interupted sutures.

The "Maison Royal de Sante" is a small hospital, situated on Rue St. Martin. Marjolin is one of the consulting surgeons.

"Beaujon" is at the extreme west of the town, and contains 400 beds. Louis is one of the physicians, and among the attending surgeons are Marjolin and Robert.

On my way to the Military Hospital de Picpus, at No. 15 Rue de Picpus, I visited the small cemetery which contains the tomb of Lafayette, a spot sacred to all Americans. He lies with his family in the farther end of the cemetery. Two plain black slabs, placed side and side, cover the remains of himself and wife. Lafayette is dead, and his great surgeon Boyer is dead—so let them rest. In 1803 Lafayette broke his thigh, and Boyer reduced it with his straight splint. But the extension requisite to prevent displacement was so great that on the twentieth day, when Boyer examined the limb, a deep ulceration had occurred on the instep and in the groin, exposing in the latter place the femoral artery! and a contraction of the muscles about the hip joint followed, producing a lameness which continued the remainder of his life. Yet Boyer never thought himself to blame, considering the ulceration as only an evidence of the perfection of his instrument, which, with its powerful screw, enabled him to make most complete extension; and if he thereby produced ulceration, he did also prevent shortening, which is generally regarded as the greater evil. Both these men were first in their day, and both we think may have done some bad surgery as well as much good. The Republicans of France say that when Lafayette placed Louis Phillippe upon the throne he committed a most fatal mistake; and that the Citizen King turns the screw so tight, that that bands sink to the very bones, and every muscle and thong of the nation is ready to break. Lafayette knew and confessed his error before he died.

"Maison Royal de Charenton" is the great Lunatic Asylum where M. Foville practices, and is most beautifully situated near the junction of the Marne and Seine, a little out of Paris. It accomodates at present about 400 patients of both sexes, who are furnished with every species of amusement and employment, and they are treated almost without restraint.

"Bicetre," situated about a mile and half outside of the Barriere d'Italie, is an establishment similar to Saltpetriere, but devoted exclusively to males, both lunatics and infirm old men. Here the straight jacket is occasionally employed. It is furnished with a pleasant garden and several fine courts, where the inmates are permitted to walk. Malgaigne, Nelaton, Moreau and Heurteloup are among the medical officers.

BELGIUM.

At half-past five, A. M. I answered to the call of the "conducteur," "No. 2, Banquette," and took my place accordingly, and at six o'clock we sallied out of the narrow gateway of the "Messagerie" into Rue St. Honore, and from thence through a labarynth of narrow and zigzag streets, to avoid the crowd which begins thus early to block up St. Denis. Crack, crack, crack goes simultaneously, right and left, the long whip of the driver and the short whip of the postillion—the "conducteur" shouts incessantly—the horses clatter, and the heavy lumbering diligence knocks and thunders along. Such a noise—such a terrible din—such a screaming of grisettes nearly run over—such a cursing of draymen in blouses, with their carts overturned—I shall never witness again until I get back to Paris—when that will be I will not say—perhaps soon—perhaps never. So adieu, belle ville!

There are many queer old walled towns between Paris and Brussels, which are so old and gloomy and fierce-looking that I almost shudder now to think of them. Peronne on the Somme is a kind of Gibralter, and contains the strong castle in which Charles the Simple, as far back as the 10th century, was imprisoned and died. The whole town looks like a prison, and if it was as strong in 1815 as now, the French, when retreating from Waterloo, ought to have made here a better resistance than they did. Cambray (hence *Cambric*) was the residence of Fenelon.

At Valenciennes, the frontier town of Belgium, we took the railroad now completed to Brussels, passing through Conde, Mons—founded by Julius Cæsar, Soignies, Hal and other minor towns.

Brussels has become since the last revolution a miniature Paris, having its boulevards, its champs elysees, its cafes, its restaurants, its foundling hospitals, and its morgue. But it has other and more interesting associations to signalize it than its resemblance to Paris.

It has been the theatre of many memorable and bloody scenes under its Brabantine, Flemish, Spanish, Dutch and French sovereigns. We remember also that it was the birth place of Vanhelmont, the chemist and physician; and of Vesalius, the anatomist, who nearly lost his life on the rack for having opened a body so soon after death as that the heart was by the stimulus of the wounds made to palpitate. That, however, which most of all must ever hereafter distinguish Brussels, is its vicinity to the field of Waterloo.

The hospital of St. Peter, situated near the Hal gate, was originally a hospital for lepers, and for wounded soldiers returning from the holy wars. It is now used as a general hospital. Portions of it are fittted up for pay patients in a very tasty and comfortable manner. M. Suetin, Professor of Surgery in the University of Brussels, is the principal surgeon to St. Pierre; and here I was permitted to see and examine carefully several cases of fractured limbs, which were now or had latety been under treatment by his starch bandage, or the *apparatus immobile.* I saw the bandage applied in one case of fractured tibia and fibula. But I have seen nothing to change my opinion, long entertained, that the cases in which either starch, dextrine, or any similar immovable apparatus can be used with propriety are comparatively few. There is also connected with this hospital an opthalmic department, which I was told was under the charge of M. Cunier, whose writings and reports frequently find their way into American journals.

After a day spent in surveying the field of Waterloo, under the guidance of Mr. Cotton, who was a sergeant-major in the Seventh Regiment of Hussars under Wellington, I pushed on through Mechlin—famed for its lace, its cathedral, its beer and its gingerbread—to Antwerp.

---

Dr. Flint :—I have drawn up and had executed carefully, an outline map of the principal Hospitals, Alms-Houses, &c., in Paris, with such references as will, I think, enable your readers easily to recognise their respective locations, which I send you for the use of your Journal. I will send you a similar map of the Hospitals of London.

Yours truly,

F. H. HAMILTON.

## Figures and Tables

**Figure f1:**